# Extract from a Treatise on Second Dentition

**Published:** 1839-06

**Authors:** C. F. Delabarre, C.A. Harris

**Affiliations:** Surgeon Dentist.


*Extract from a Treatise on Second Dentition, by C. F. Delabarre, Translated by C. A. Harris, M. D. Surgeon Dentist.**

### GENERAL CONSIDERATIONS.

"Whilst the various branches of the healing art, are fast approaching to perfection, and the light of pathological research now lends its aid to practitioners, in the most complicated diseases; Dentistry by no means

---

* On Second Dentition, this is the best if not the only complete work extant; but it has for a long time remained either wholly unknown to practitioners, or they have only resorted to it as a work of refference, without being able to devote sufficient time to master the difficulties of a foreign tongue, in order thus to fully understand keeps pace with these improvements. It does not receive that attention from public schools, which its importance demands; and although we have many works, ex-professo, on the structure and diseases of the teeth, yet many more are still to be desired. Authors on Nosology and Physiology usually confine themselves to some particular department of medicine, and discuss in but a very superficial manner, all other subjects, which for the completion of their works they are compelled to notice. Many of them, indeed, though fully confessing the great want of knowledge that exists, of the expansion, growth and affections of the teeth, are nevertheless, unable, by reason of their numerous engagements, to devote them selves to this study.

These bones, indeed, were formerly regarded merely as ornaments of the face, and accordingly their preservation was rather the province of the perfumer than of the regular physician.

But modern physiologists, in lecturing on the phenomena of digestion, have now very clearly shown, in what manner the teeth are necessary for the performance of one of those functions on which life depends. It is now well known that the alimentary canal is charged with those substances designed for growth and preservation of the individual, and it must be aided in its operations by mechanical means. Man's stomach cannot act with ease, unless the destined aliment has first passed through a previous preparation. Accordingly, our food is first roasted or boiled and seasoned in various ways before it is committed to the mouth, in order that it may the better be prepared to be submitted to the action of the stomach, by its being penetrated with saliva, the secretion of which is called forth by the presence of the food, and by mastication. But how can this last be performed with jaws unfurnished with teeth?

In those animals which have a membranous stomach, the teeth are designed as auxiliaries to facilitate its operations; and, as it has pleased the Creator to use an endless variety of means to produce the same results, those species, whose stomachs are of a weak texture, are supplied with teeth, while those which possess this organ endowed with great muscular power are unfurnished with them.

The stomach in animals of the ruminating kind, whose dental arch is incomplete, generally supplies the want of these bones, and the food is brought again to the mouth, there to be masticated anew and to be submitted again to *insalivation.*

The physicians of our day, better appreciate the importance of Dentistry than those of a former age; they recommend it to our attention,—they do not neglect to examine the habitual healthy state of the mouth, and hence, they frequently make inductions which are useful to them in cases of disease.*

The phenomena exhibited in the development of these small bones, which now engage our notice, have attracted the attention of some, and

---

its contents. A treatise on this part of the science, has long been needed in our own language; and with a view of supplying this want, the translator contemplates presenting in a short time, the whole work to the American public, accompanied with numerous notes and an introduction. HARRIS.

* Notwithstanding the excellence of the most modern treatises on semiology, there are a certain number of important symptoms connected with the jaws, the saliva, the tartar, &c. which have not yet been mentioned.

excited their curiosity; but, to speak freely, this part of the science is still too much neglected and too little understood,—it demands a less superficial study, from those who devote themselves to the honourable task of instructing their pupils in all the branches of the great medical art.

Although there have been a great many very interesting works on First Dentition, still all the phenomena of which it is composed, have not yet been fully enumerated; it is for time and experience to supply these deficiencies.—Happy will he be who shall diminish their number.*

First Dentition, however, has been under the inspection of men of approved medical knowledge; it is not thus with the branch we are now considering;—it has been left to the observation and sagacity of Dentists, possessing none of the knowledge which is to be found only in physiology.

The laws that govern the expansion, growth and arrangement of the teeth, are properly the patrimony of the physician, who should understand those, in order to direct the dentist whenever (as is sometimes unfortunately the case,) he is not furnished with sufficient information on all the duties of his profession.

It is very desirable that there should be a class of physicians, who give their attention to the practice for diseases of the mouth; this is the only means of preventing so important a part of our art from falling into the unskilful hands of mere practitioners, or from becoming the prey of the vilest charlatans. Such persons cannot practice surgery without detriment to society.

He that is nothing more than a mechanician, ought not to be admitted into the sanctuary of Esculapius. His duty here, should be confined to the management of those machines, with which alone he has the right to interfere.†

I repeat it, the knowledge of true Dentistry is not sufficiently diffused, and never will be, until it shall have been made as much a portion of public instruction, as the various other parts of the art; then, and then only will it be cultivated by educated men, and advance with the same vigour as the other branches of medicine.

The management of Second Dentition, is a stumbling-block to Dentists of moderate abilities; they have for this, a certain routine, which, I say it with regret, is sustained by writings of authority with those who find in these books of ready made opinions, food for their minds, and foundation for their practice, without giving themselves the trouble to estimate their value.

It is in order to eradicate these errors, that I have determined to pub-

---

* The work of Professor Beaumes, on First Dentition, should, nevertheless, be well studied: it is justly placed in the first rank.

† I have not room here to express my regret, that goldsmiths, jewellers, watch-makers and workers in ivory, having laboured only as such in the laboratories of dentists, without having studied, without ever assisting at their operations, should obtain permission to practice. Let us however, hope, that under the wise regulations that are proposed for the practice of medicine, these sorts of admissions will no longer occur; and that with these, will also disappear those great teeth of varnished ivory that indicate the abodes of these dentists, and which to the shame of the art, are also seen at the doors of some more respectable personages.

lish this work. It is not my intention to establish any new system; the natural method which I here explain has been followed by many distinguished Dentists ; but they have either not made public the results of their experience, or have done it in a brief manner. I here show that which enlightened men practice daily with success ; and I only add some few reflections, drawn from my practice, and from an anatomical examination of the jaw, considered from the first moment that the adult teeth appear, until the period they take their place in that circle, which, after their growth they are destined to occupy. Thus submitting my observations to the just criticism of professional men, I leave it to their abilities to decide upon a subject of so much importance as to interest every class of the community.

Among the numerous authors, both native and foreign, that I have been obliged to consult before writing,* I have not found any who do not seemingly present some system founded on their own personal observation; they abandon themselves to reasonings more or less specious, and forsake the paths of experience to follow the vagaries of their imaginations. Others do not fully explain themselves on this point; they elude and seem afraid to grapple with the subject.

Sprengel has justly remarked, that the road of experience is very difficult to travel, for it is easier to follow the suggestions of fancy, and by an unvaried routine, to acquire a certain amount of skill, than to confine one's self to the narrow and thorny path of observation.

These principles may be applied to the treatment of Second Dentition; it is much easier to extract the teeth, than to determine whether this is absolutely necessary. The extraction of a tooth requires nothing more on the part of the practitioner, than a certain degree of facility in the use of the instruments that are usually employed in this operation; whilst the knowledge necessary to appreciate the consequence, can only be acquired by time and study.

Fauchard, Jourdain, Bourdet and other writers of a more modern date, have treated this point with a sort of indifference, unfortunate for the profession, and painful to him who expects to find in the works of these learned Dentists, something which at least will instruct him.

In his *Dentiste de la Jeunesse*, so finely written, M. Duval has shown great literary talents ; but this work seems rather to be designed for the public at large than for men of the profession. The author, believing that his professional brethren were as learned as he, has not taught them precisely what he should have known so well how to teach; he has not mentioned how very necessary it is in order to assist Dentition, that the means should be varied according to circumstances.

In another work, excellent in many respects, he has not reckoned among the dangers of extraction, the imperfect configuration of the Dental Arch and the destruction of the germs of adult teeth, the common result of an improper extraction, and which a practitioner of his merit could not have failed to observe."

---

* I have employed some moments of leisure to translate the works of Hunter and Fox upon the human teeth. My intention at first, was to publish them : but I have found in them so many opinions, and so many things which do not accord with our actual knowledge of physiology, that I regard them only as good sources from which we may derive information, but whose engravings constitute their greatest merit.

We do not wish to be considered as coinciding with M. Delabarre in all his views, though on the whole, we regard his Treatise on Second Dentition, as decidedly the best that has ever been published. He has, it is true, in many places, while attempting to point out errors in others, fallen into very palpable ones himself, which, should we, as it is our intention soon to do, ever publish the whole work, we propose to notice. He has, as must be acknowledged by every one acquainted with his work, discussed the subject on which it treats, in a very able and masterly manner.

The general principles laid down by Fox, for the treatment of Second Dentition, evince a thorough knowledge of the subject, and have constituted the basis of the established practice ever since, but he did not enter sufficiently into detail. There are many things connected with this subject, which are necessary for the practitioner to understand, unnoticed by him. Upon these M. Delabarre has treated at large.

